# Tailoring silk fibroin hydrophilicity and physicochemical properties using sugar alcohols for medical device coatings

**DOI:** 10.1038/s41598-024-64450-5

**Published:** 2024-06-14

**Authors:** Supranee Kaewpirom, Sarayoot Piboonnithikasem, Pongsathorn Sroisroemsap, Sittichai Uttoom, Siridech Boonsang

**Affiliations:** 1 Department of Chemistry, Faculty of Science, Burapha University, Chonburi, 20131 Thailand; 2 Department of Medical Science, National Institute of Health, Ministry of Public Health, Nonthaburi, 11000 Thailand; 3 Department of Electrical Engineering, Faculty of Engineering, King Mongkut’s Institute of Technology Ladkrabang, Bangkok, 10520 Thailand

**Keywords:** Silk fibroin, Sugar alcohol, Molecular docking, Hydrophilic coating, Water contact angle, Chemistry, Materials science

## Abstract

This study explores the modification of silk fibroin films for hydrophilic coating applications using various sugar alcohols. Films, prepared via solvent casting, incorporated glycerol, sorbitol, and maltitol, revealing distinctive transparency and UV absorption characteristics based on sugar alcohol chemical structures. X-ray diffraction confirmed a silk I to silk II transition influenced by sugar alcohols. Glycerol proved most effective in enhancing the β-sheet structure. The study also elucidated a conformational shift towards a β-sheet structure induced by sugar alcohols. Silk fibroin–sugar alcohol blind docking and sugar alcohol-sugar alcohol blind docking investigations were conducted utilizing the HDOCK Server. The computer simulation unveiled the significance of size and hydrogen bonding characteristics inherent in sugar alcohols, emphasizing their pivotal role in influencing interactions within silk fibroin matrices. Hydrophilicity of ozonized silicone surfaces improved through successful coating with silk fibroin films, particularly glycerol-containing ones, resulting in reduced contact angles. Strong adhesion between silk fibroin films and ozonized silicone surfaces was evident, indicating robust hydrogen bonding interactions. This comprehensive research provides crucial insights into sugar alcohols’ potential to modify silk fibroin film crystalline structures, offering valuable guidance for optimizing their design and functionality, especially in silicone coating applications.

## Introduction

Hydrophilic coatings are acknowledged for their ability to render medical devices wettable while minimizing surface friction. Amongst these materials, our prior study has demonstrated that modified regenerated silk fibroin (SF) stands out as a promising choice for hydrophilic coating applications^1^. This is attributed to its remarkable characteristics, including high biocompatibility, excellent biodegradability, and its absence of immunogenicity and allergenicity. There are diverse approaches for treating silk fibroin to enhance its characteristics, specifically emphasizing the improvement of its mechanical properties by increasing the degree of crystallinity. According to Kweon et al.^2^, the primary conformation of silk fibroin is characterized by a random α-helix coil, which exhibits instability in water. When subjected to treatment with aqueous methanol^1,3^, a transition occurs, leading to a more stable secondary structure identified as the β-sheet. This transition contributes to heightened modulus and tensile strength; nevertheless, this enhanced strength is accompanied by increased brittleness.

To address this limitation, certain plasticizers, exemplified by glycerol, have been utilized to augment the stretchability and toughness of silk films^1,4^. Through this approach, it becomes feasible to regulating the molecular assembly of the silk proteins by incorporating glycerol. The suggestion has been made that glycerol might concurrently instigate and impede the formation of β-sheet structures in silk materials, leading to some irregular folding and subsequently yielding less organized silk II structures^5^. The utilization of glycerol as a plasticizer demonstrated a notable enhancement in the ductility of composite films based on silk fibroin^6^. The inclusion of glycerol as a plasticizer molecule results in a reduction of intermolecular forces along the polymer chains. This, in turn, enhances the mobility and distensibility of the biopolymer matrix while simultaneously increasing chain extensibility and resistance to fracture.

Sugar alcohols are acknowledged as the predominantly utilized plasticizers, imparting a more significant plasticizing effect to hydrocolloid-based edible films. They are frequently employed as natural-derived plasticizers in conjunction with hydrophilic biopolymers such as starch^7^, oxidized nanocellulose^8^, soy protein-based bioplastics^9^, and quince seed gum-alginate beads^10^ Furthermore, several of these sugar alcohols have gained international approval for inclusion in food products. This characteristic renders them well-suited for potential utilization in the development of edible, biodegradable, biocompatible, and hydrophilic films. However, the efficacy of individual plasticizers is linked to factors such as molecular size, shape, the presence of free hydroxyl groups, arrangement of oxygen atoms, water-binding capacity, and the configuration of the biopolymer. This includes the compatibility of the plasticizer with the film-forming polymer to ensure homogeneous distribution within the three-dimensional structure of the film^7^.

While the fabrication of silk fibroin/glycerol composites has been previously reported by our group^1^ and others^6,11^, the incorporation of silk fibroin with various sugar alcohols to alter SF film properties is not widely documented. Therefore, we aim to demonstrate the potential application of SF films as hydrophilic coatings for medical devices to enhance their functionality. Many medical devices that come into contact with body fluids, blood, and tissue—such as tubes, catheters, mechanical heart valves, lenses, and disposable plastic slides—require improved wettability to function effectively with physiological fluids and tissues. Modified regenerated SF is considered a promising material for hydrophilic coatings due to its exceptional mechanical properties, high biocompatibility, superior biodegradability, and lack of immunogenicity and allergenicity.

In this context, it is imperative to investigate a range of plasticizers to elucidate their effectiveness in formulating hydrophilic coatings. As of our current knowledge, there is a notable absence of dedicated studies examining the impact of different sugar alcohol plasticizers on the properties of silk fibroin coatings. Previous research has well established that β-sheet crystallites significantly influence the properties of silk fibroin materials^12^ Numerous researchers have employed FTIR to investigate the conformation transition kinetics from random coil and/or helix to β-sheet in silk proteins when exposed to various solvents, including methanol, ethanol, propanol, and isopropanol^1,12–14^ In our previous study^15^, it was demonstrated that alcohol treatment reduced the β-sheet content in silk fibroin films. However, in this current study, it is expected that treatment with sugar alcohols will enhance the crystallization of the films, thereby increasing the β-sheet content. This effect varies depending on the chemical structure of the sugar alcohols and their interaction with the silk protein chains. Through computational modeling, the interactions between silk protein chains and sugar alcohols are elucidated. This underscores the significance and novelty of the present study, which employs protein homology modeling and molecular docking to clarify these interactions, thereby promoting β-sheet formation that influences the properties of silk films.

Therefore, the primary objective of this investigation was to thoroughly examine the hydrophilicity and physicochemical properties of silk fibroin coatings. This exploration specifically focused on the influence of sugar alcohol plasticizers with varying carbon atoms and hydroxyl groups. The computer simulation was also employed to elucidate the interactions between sugar alcohol molecules and silk protein chains, as well as the interactions among the sugar alcohol molecules themselves. Additionally, the study aimed to assess the impact of these plasticizers on the microstructure, water contact angle, and cross-cut adhesion of hydrophilic silk fibroin coatings on a silicone surface that had undergone hydrophilization through UV/Ozone treatment.

## Materials and methods

### Materials

White *Bombyx mori* cocoons were from Udon Thani province, Thailand. Na_2_CO_3_ and CaCl_2_ were bought from Ajax Finechem, and ethanol was purchased from QREC. Sugar alcohols, specifically glycerol, sorbitol, and maltitol, were procured from Chemipan Corporation Co., Ltd. The chemicals employed in the study were of analytical grade and utilized without any additional purification. Silicone sheet (commercial grade) with a thickness of approximately 1.5 mm was purchased from Srcaster, Thailand.

### Preparation of SF solution

The extraction of SF was accomplished using a standard degumming procedure, which entailed boiling 2.5 g of finely chopped silk cocoons in a one-liter aqueous solution of 0.02 M Na_2_CO_3_ for 30 min, with intermittent stirring. After degumming, the silk fibroin was rinsed with distilled water until it reached a neutral pH. It was then dissolved in a solution of CaCl_2_, C_2_H_5_OH, and H_2_O with a mole ratio of 1:2:8, at 110 °C for 2 h. Following this, the salt was eliminated via dialysis against deionized water at room temperature (30–32 °C) for a total duration of 72 h. A cellulose dialysis bag with a molecular cut-off (MWCO) of 12–14 KDa (Cellu Sep®) was employed for this process. The resultant suspension of silk fibroin was subsequently subjected to purification through centrifugation at 2200 rpm for 20 min at room temperature, effectively eliminating impurities. The homogeneous silk fibroin solution's final concentration, determined using a gravimetric method, was approximately 5.0% w/v.

To alter the properties of silk films, following the production of the initial silk fibroin solution, sugar alcohols, namely glycerol, sorbitol, and maltitol, were incorporated into the purified homogeneous silk fibroin solution at a concentration of 30%w/w relative to the dry weight of the silk fibroin film before the film formation process. Chemical structure, numbers of C atom, and numbers of OH group present in the sugar alcohols incorporated into silk fibroin films are presented in Supplementary Table S1 online.

### Preparation of SF film

The formation of distinct silk fibroin films was achieved by pouring a 5% w/v silk fibroin solution (7.5 mL) into polyethylene terephthalate molds and allowing it to air-dry at room temperature for 48 h, both with and without the inclusion of sugar alcohols. To remove any remaining water molecules, the drying process was reiterated in a vacuum oven at room temperature for an additional 4 h. Films of silk fibroin with modified properties were generated, featuring thicknesses of approximately 50 µm.

### SF coating on silicone surface

Before the coating process, silicone sheets measuring 25.4 mm × 76 mm × 1.5 mm underwent ultraviolet/ozone treatment using a commercial UV/ozone chamber. This chamber utilizes a low-pressure grid mercury lamp to generate UVO radiation with a primary emission in the ultraviolet range, facilitated by a fused quartz UV lamp with approximately 65% transmission at 284 nm. To optimize the exposure of the treated surface to radiation, all silicone sheets were positioned 3 cm away from the lamp and exposed for a duration of 1 h. During the coating procedure, the silk fibroin solution, with or without the supplementary sugar alcohols, was applied onto the ozone-treated silicone sheets using a bar-coater equipped with rod No. 2 (K Hand coater, RK Printcoat Instrument, UK). Subsequently, the coated sheets were left to air-dry at room temperature for a period of 24 h. A silk fibroin film with a thickness of 10 µm was successfully produced.

### Structural analysis using FTIR

Analysis of the chemical structure of the silk fibroin films was conducted by examining the functional groups using an FT-IR spectrophotometer (PerkinElmer Frontier™ FT-IR/NIR system) equipped with a multiple-reflection ATR attachment. In each measurement, 4 scans were combined with a resolution of 4 cm^−1^, and wavenumber ranged from 400 to 4000 cm^−1^.

For a more detailed analysis, curve-fitting of the Amide I region was employed in this study using a Python library^16^. The peak positions of the fitted curves were assigned to their potential secondary structure conformations and side chains, as detailed in our previous study^15^. This approach not only enhances the precision of structural analysis but also reduces the subjectivity typically associated with manual peak fitting.

### Wide angle X-ray diffraction (WAXD) analysis

WAXD analysis was conducted using a versatile X-ray diffraction system (Rigaku model: SmartLab SE) operating at 40 kV and 30 mA, employing Cu-Kα radiation. The diffraction intensity was captured in reflection mode at a scanning speed of 2°/min with a diffraction angle (2θ) range of 5 to 90°.

### Insoluble gel fraction

The gel fraction of the silk fibroin film was assessed using a gravimetric method. Before conducting the analysis, the silk fibroin film, with an area of 3.0 × 3.0 cm^2^, was dried at 60 °C for 24 h and its weight (W_d_) was measured. Subsequently, the dried film was immersed in deionized water at room temperature (29 °C) for 24 h to remove the soluble portion. The solid film that remained was then dried at 100 °C for 2 h and its weight (W_rd_) was recorded. The gel fraction was determined by calculating the percentage ratio of the weight of the remaining solid to the initial weight of the dried film, as described in Eq. (2):

2 $$Gel\; fraction (\%)=\frac{{W}_{rd}}{{W}_{d}}\times 100$$

### Moisture content

The moisture content was determined by assessing the weight loss of the films (ISO 15,512). SF films were cut into square pieces of 4.0 × 4.0 cm^2^ and weighed precisely (W_m_). The dry mass of the films was recorded after drying them in an oven at 110 °C for 4 h or until a constant weight (W_d_) was achieved. The moisture content measurement was repeated three times for each film treatment. The moisture content (%) was calculated using the following formula:

3 $$ Moisture \,content \left( \% \right) = \frac{{\left( {W_{m} - W_{d} } \right)}}{{W_{d} }} \times 100 $$

### Water contact angle

Water contact angle (WCA) measurements were conducted by placing an 8 µL DI water droplet on the surface of the silk fibroin film, and side-view images of the droplet were captured. To minimize the impact of air-gaps, a drop of water was suspended from a needle, and then the film surface was carefully brought into contact with this hanging droplet. Each reported WCA value represented the mean result derived from a minimum of five measurements, obtained from various locations on the silk fibroin film.

### Cross-cut adhesion

The adhesion strength of the silk fibroin films was evaluated following the ASTM D-3359-97 (Method B) standard. Square boxes measuring 1 mm^2^ were created on a 1 cm × 1 cm area of the test specimen. These squares were then covered with 3 M™ adhesive tape. Subsequently, the tape was peeled off, and the count of the number of boxes that were detached from the surface was used to assess the adhesion strength of the films. To assess the removal of the silk fibroin coating from the silicone surface, the grid area was examined under an illuminated magnifier. The adhesion strength was subsequently rated on a scale ranging from 0 to 5B in accordance with the following scale: 5B = no removal, 4B = less than 5% removal, 3B = 5 to 15% removal, 2B = 15 to 35% removal, 1B = 35 to 65% removal and 0B = more than 65% removal.

### Scanning electron microscopy

The surface and cross-sectional structures of the films were analyzed through scanning electron microscopy (SEM). For sample preparation, the dried films were affixed to metal stubs using double-sided adhesive carbon tape. Following that, a thin layer of gold was applied to the film surfaces through a sputter-coating procedure using the Q150R ES system from Quorum Technologies, England. SEM images of the films were obtained using an LEO-1450VP scanning electron microscope, which operated at an accelerating voltage of 15 kV.

### Computer modelling

The three-dimensional configuration of silk fibroin derived from *Bombyx mori* was reconstructed through the utilization of two computational modeling servers: (1) The Swiss Model Workspace Server, accessible at https://swissmodel.expasy.org/interactive,^17^ and (2) The trRosetta server, accessible at https://yanglab.qd.sdu.edu.cn/trRosetta.^18^ The primary structure of the Fibroin light chain [*Bombyx mandarina*] protein, with accession numbers XP_028029832, was acquired from the Entrez Molecular Sequence Database System from accession numbers XP_028029832, accessible at https://www.ncbi.nlm.nih.gov /Web/Search/entrezfs.html. Validation of the computational model was conducted by assessing each server algorithm. The AlphaFold accession number P21828, specifically the AF-P21828-F1-model-v4_P21828_FIBL_BOMMO_Bombyx mori_Silk moth, available at https://alphafold.ebi.ac.uk/entry/P21828, served as the template for the computational modeling process. Subsequently, root-mean-square deviation (RMSD) values were calculated through superimposition with the aforementioned template.

Protein–ligand blind docking and ligand-ligand blind docking investigations were conducted utilizing the HDOCK Server, a freely accessible computational docking server, which can be accessed at http://hdock.phys.hust.edu.cn/.^19^ The interaction between the fibroin light chain and ligands, as well as the self-interaction among ligands, was evaluated through computational docking utilizing the HDOCK server. The tertiary structure of the fibroin light chain, derived from the reference sequence XP_028029832 with the smallest root-mean-square deviation (RMSD), was employed as the protein target. Subsequently, this protein target was subjected to docking with three ligands—glycerol (Glycerol_CID_753), D-Sorbitol (D-Sorbitol_CID_5780), and D-Maltitol (D-Maltitol_CID_493591). The molecular structures of all three ligands were retrieved from the PubChem Database (https://pubchem.ncbi.nlm.nih.gov/). To facilitate comprehensive comparison, all docking results were superimposed onto the same protein target, thereby consolidating the outcomes into a singular structure for comparative analysis.

## Results and discussion

### Physical appearance and structural characterization

**Figure 1:**
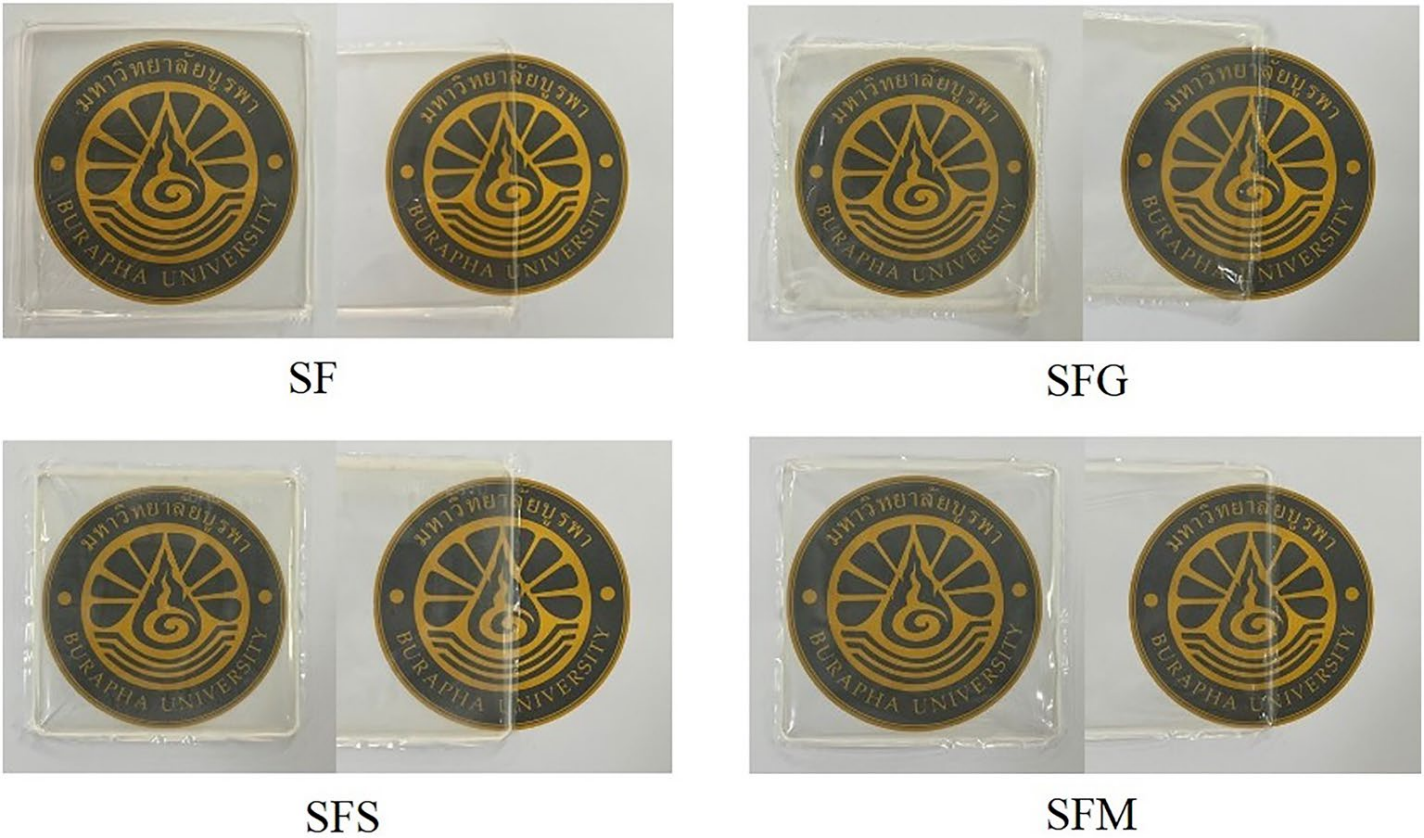
Silk fibroin films obtained by casting method. The pure silk fibroin film is denoted as SF, while the silk fibroin films containing glycerol, sorbitol, and maltitol are identified as SFG, SFS, and SFM, respectively.

Silk fibroin has gained prominence in the biomedical domain due to its exceptional mechanical attributes, compatibility with biological systems, minimal potential for causing inflammatory responses, and extended biodegradability over time. Moreover, it is a straightforward process to transform regenerated silk fibroin solutions into film-like structures. Plasticizers like glycerol can be utilized to modify the properties of silk fibroin films by integrating a glycerol solution into the film fabrication process^1^. Fig. 1 illustrates various formulations of silk fibroin films achieved through the casting method. In this research, sugar alcohols with diverse chemical structures were utilized to modify the characteristics of the films. It is evident from the figure that all the films prepared exhibit distinct transparency and UV absorption characteristics. The films demonstrate transmittance percentages of 6% to 16% at 250 nm and 87% to 90% at 550 nm, depending on the type of sugar alcohols incorporated (see Supplementary Fig. S1 online).

**Figure 2:**
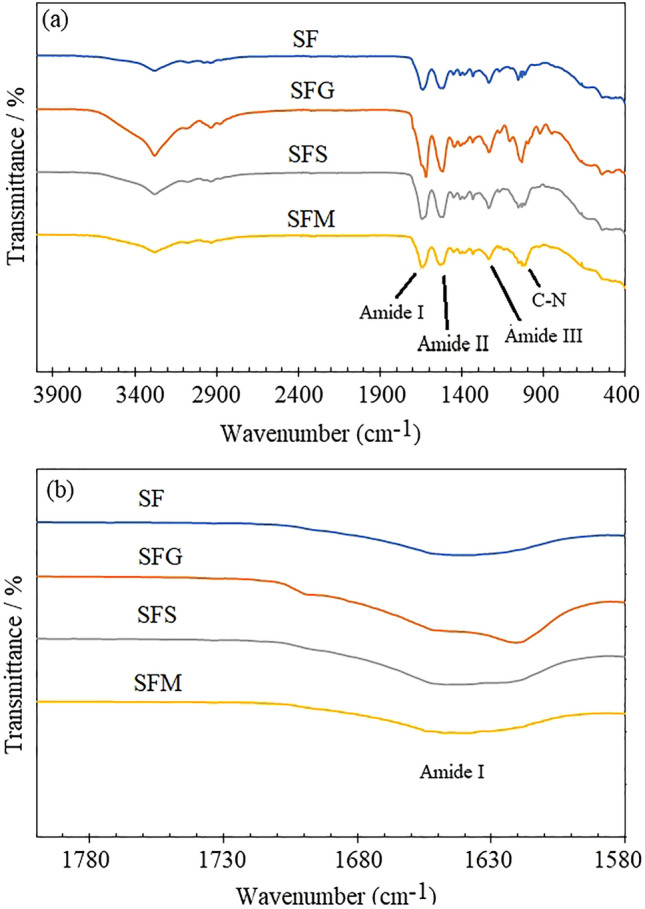
Fourier-transform infrared spectroscopy (FTIR) spectra of silk fibroin films, exhibiting alterations in peak intensity within the range of 1580 to 1730 cm^−1^.

The investigation of structural changes in silk fibroin due to the application of sugar alcohols was also conducted using Fourier-transform infrared spectroscopy (FTIR), and the findings are presented in Fig. 2. All the silk films exhibit similar peaks that are characteristic of silk fibroin. In Fig. 2a, the broad absorption signal spanning from 3100 to 3700 cm^−1^, coupled with a distinct peak at 3277 cm^−1^, can be attributed to the stretching vibration of N−H and O−H within peptide groups, along with hydrogen bonding within the silk fibroin structure. The peaks observed at 3075, 2935, and 2872 cm^−1^ correspond to C−H aromatic, C−H asymmetrical, and C−H symmetrical vibrations, respectively. The peaks associated with peptides observed at 1640, 1525, and 1237 cm^−1^ have been assigned to amide I (C=O stretching, β-sheet), amide II (C−N stretching and N−H bending, β-sheet), and amide III (C−N stretching, C−O bending vibration, alpha-helix/random coils), respectively^15,20,21^. Upon the addition of sugar alcohols, the characteristic absorption bands of amide I, amide II, and amide III exhibited no significant shifts in peak positions, but there was a notable increase in peak intensities. A broadening of the signals within the 3100 to 3700 cm^−1^ range, attributed to specific interactions between sugar alcohols and silk molecules, was also noted. The substitution of silk molecule-silk molecule interactions with silk molecule-sugar alcohol plasticizer interactions occurred as a result of the hydroxyl groups in the silk molecules contributing to the hydroxyl group of the sugar alcohol. These interactions had an impact on the crystallization behavior of the films^11^. This indicates that the silk molecules in the films with added sugar alcohols formed an insoluble β-sheet structure. Additionally, there was a widening and separation of the amide I band. The peak at 1623 cm^−1^, which corresponds to the β-sheet structure, exhibited greater prominence (Fig. 2b).

It’s worth noting that the impact of the chemical composition of sugar alcohols on the crystallization of silk fibroin films was notably observed through the widening of signals in the spectral ranges of 3100 to 3700 cm^−1^ and of 1580 − 1730 cm^−1^. Among these, glycerol exhibited the highest degree of signal broadening, signifying its remarkable efficacy as a plasticizer capable of modifying the crystal structure of silk fibroin by promoting an enhanced β-sheet structure. As the number of carbon atoms and hydrogen groups increased, the extent of signal broadening decreased. Consequently, in terms of their effectiveness as sugar alcohol plasticizers for enhancing the β-sheet structure, the ranking is as follows: glycerol > sorbitol > maltitol. The reason for these is that the small size of glycerol molecule improves its efficiency as a plasticizer and brought about more direct interactions between polymer molecules^7^. The increased number of hydroxyl groups is associated with larger sugar alcohols, and it appears that the structural characteristics of sorbitol and maltitol, particularly their ring structure, limits their ability to reduce hydrogen bonding between the polymer chains^7^. In conclusion, it is evident that sugar alcohols have the potential to modify the crystalline structure of silk fibroin films. When evaluating their effectiveness in promoting the β-sheet structure, the ranking is as follows: glycerol > sorbitol > maltitol.

**Figure 3:**
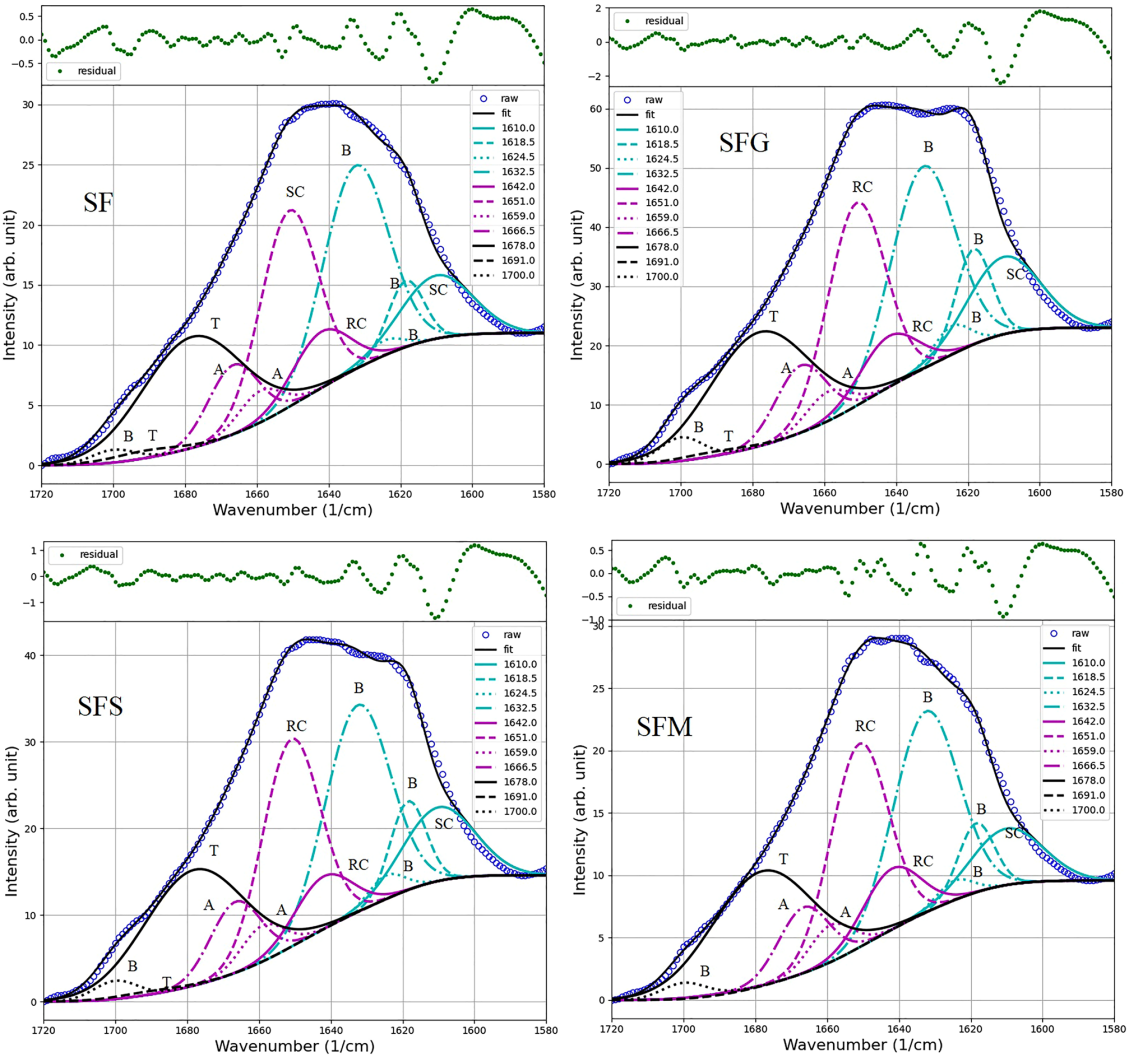
The absorbance Amide I spectra of silk fibroin films without alcohol treatment (SF), and treated with glycerol (SFG), sorbitol (SFS), and maltitol (SFM). The spectra are presented after fitting with a Gaussian profile. The solid line represents the resulting absorbance band, while the dotted lines indicate the contributions to the Amide I band, labeled as random coil (R), β-sheets (B), alpha-helices (A), beta-turns (T), and side chains (SC).

To quantify the β-sheet content in silk fibroin films treated with sugar alcohols, curve-fitting of the Amide I region was employed in this study using a Python library. This method was utilized to examine the conformational structural content of the silk fibroin films. Qiu et al.^14^ have similarly noted that the β-crystallites and the interactions among helical nanofibrils are two of the most crucial structural elements that significantly influence the macroscopic performance of various forms of silk materials. Figure 3 presents the fitted results for SF, the silk fibroin film without sugar alcohol treatment. It reveals the distinctive β-sheet characteristics at 1632.5 cm^−1^and 1618.5 cm^−1^, along with two weak peaks at 1624.5 cm^−1^and 1700 cm^−1^. Upon the addition of sugar alcohols, the peaks characteristic of β-sheets increased significantly for SFG and SFS, with relative contents of 1.0 and 0.7 a.u., respectively. In contrast, SFM exhibited the lowest β-sheet content of 0.4 a.u. (see Supplementary Fig. S2 online). Thus, it is affirmed that sugar alcohols possess the capability to alter the crystalline structure of silk fibroin films. When assessing their efficacy in enhancing the β-sheet structure, the hierarchy is as follows: glycerol > sorbitol > maltitol.

Further investigation of the crystallization structure associated with β-sheet formation was conducted through the utilization of the wide-angle X-ray diffraction technique. Subsequent exploration of the interactions between silk fibroin chains and sugar alcohol molecules will be expounded upon in the following section, employing protein structure homology modeling.

### Wide-angle X-ray diffraction

**Figure 4:**
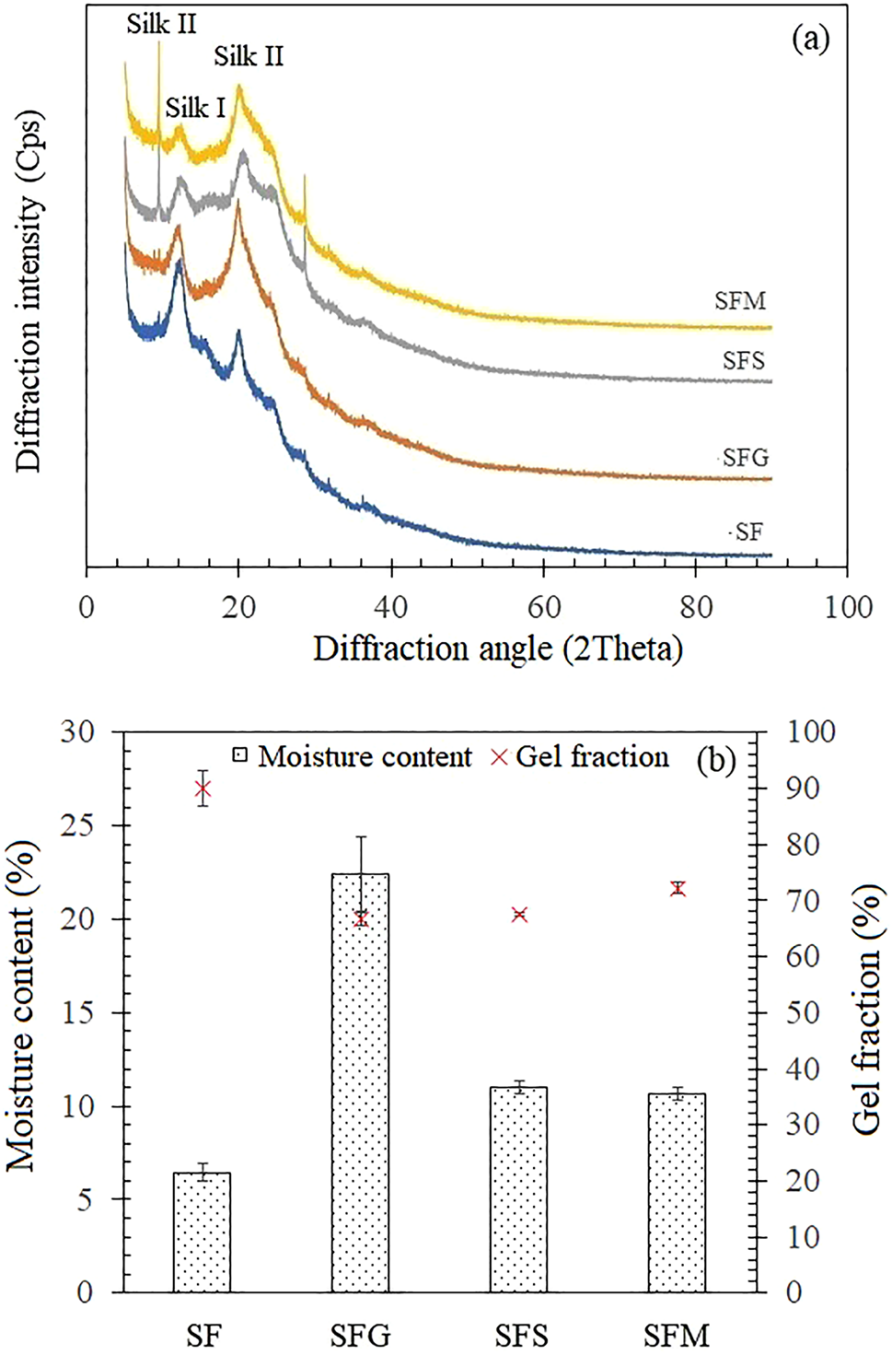
Wide-angle X-ray diffraction (WAXD) patterns (**a**) and insoluble gel fraction and moisture content (**b**) for silk fibroin films: pure silk fibroin films (SF) and silk fibroin films with the incorporation of sugar alcohols, specifically glycerol (SFG), sorbitol (SFS), and maltitol (SFM).

Figure 4a displays the XRD spectrum of the pure silk fibroin film. The peaks observed at 12.8° and 28.3° can be attributed to the presence of silk I structure, while the peaks at 9.5°, 20.1° and 24.5° correspond to silk II structure^1,22,23^. This observation suggests that the pure silk fibroin film consists of both silk I and silk II structures with a lower prevalence of the β-sheet and amorphous structural components. Upon the addition of sugar alcohols, there was a noticeable reduction in the intensity of the peaks at 12.8° and 28.3°, accompanied by an increase in the intensity of the peaks at 9.5° and 20.1°. Interestingly, the intensity of the peaks at 24.5° and 36.2° remained relatively stable. These findings collectively suggest that sugar alcohol treatment facilitates the conversion of the silk I structure into silk II. Similar results were proposed by many authors, confirming that the silk structure can be modified via methanol or potassium phosphate treatment^24^, water and alcohol treatment^15,25^, and glycerol treatment^1^. The validation of crystal structure modification was further substantiated through the determination of the relative crystallinity index. This index was computed by assessing the difference in peak intensity between the amorphous peak at 15.8° and the crystalline peak at 20.1° of silk II structure, which was then normalized by the intensity of the peak at 20.1°. Following the application of sugar alcohol, silk fibroin films containing glycerol (SFG) exhibited an elevated crystallinity index of 26.6%, surpassing that of the pristine silk fibroin film (SF) with a crystallinity index of 1.5%. The SFS demonstrated a crystallinity index of 16.6%, while SFM exhibited a crystallinity index of 23.3%.

Lu et al.^11^ proposed that the incorporation of glycerol into silk fibroin films facilitates the interaction of glycerol molecules with silk fibroin chains through intermolecular forces, particularly through the formation of hydrogen bonds between the hydroxyl groups of glycerol and the amide groups of silk. This interaction is likely to have modified the hydrophobic hydration state of the protein chains. This effect leads to a reduction in the content of α-helical structures, while increasing the content of β-sheet and β-turn structures. Consequently, this phenomenon results in the stabilization of silk II structures, characterized by a crystalline arrangement of β sheets.

The impact of sugar alcohols on the conversion of the silk I structure into silk II was notably subdued when larger sugar alcohols with an increased number of hydroxyl groups were utilized. Typically, small molecules can readily integrate with polymer chains owing to their compact size. Conversely, larger molecules possess a greater molecular weight, and their effectiveness lies in their capacity to fuse with the host polymer at the molecular level. Following the blending of silk fibroin with larger sugar alcohols and the casting of films, it was observed that the larger sugar alcohol molecules did not fuse with silk fibroin chains as effectively as glycerol did. The occurrence of the hydrophobic effect, which results in the seclusion of nonpolar side chains within the interior of proteins and subsequently leads to the compaction of polypeptide chains into compact, globular structures, was hard to achieve. As a result, the conformational transition from a random coil to α-helices, which represents an unstable intermediate state on the path toward forming stable β-sheet structures, infrequently took place.

### Gel fraction and moisture content

Figure 4b illustrates the insoluble gel fraction of silk fibroin following a 24-h immersion in deionized water at room temperature (29 °C), aiming to eliminate the soluble component.

In the absence of sugar alcohols, silk fibroin exhibits a gel fraction of 89.9 ± 3.2%. This value significantly exceeds the gel fractions observed in sugar alcohol-incorporated silk fibroin films, which are 66.7 ± 1.2% for SFG, 67.5 ± 0.4% for SFM, and 72.3 ± 1.0% for SFS. The observed low gel fraction values in silk films containing sugar alcohols correlated well with the moisture content of SF films (SFG > SFS > SFM > SF). This could be attributed to the water solubility of sugar alcohols, which ranks as follows: glycerol > sorbitol > maltitol (see Supplymentary Table S1 online).This phenomenon can be also attributed to the robust intermolecular interactions among protein chains, resulting in the water-insolubility of silk fibroin films with a significant degree of insoluble content. This finding aligns well with the research conducted by Cheng and colleagues^26^, who assert that the primary structural constituents of crystalline domains within silk fibroin are the polypeptide chains primarily composed of the amino acids glycine (Gly) and alanine (Ala). These neighboring chains are bound together by robust hydrogen bonds, adopting an anti-parallel configuration to create β-sheets. While the incorporation of sugar alcohols, especially glycerol, into silk films led to an elevation in β-sheet content in comparison to untreated silk, a portion of the random coil structure was retained. Sugar alcohols have the potential to interfere with the formation of β-sheet structures, in contrast to silk-only films. On the other hand, sugar alcohols also tend to maintain a higher proportion of disordered coil structures, leading to a reduced extent of insoluble gel fraction.

### Protein homology modelling and molecular docking

**Figure 5:**
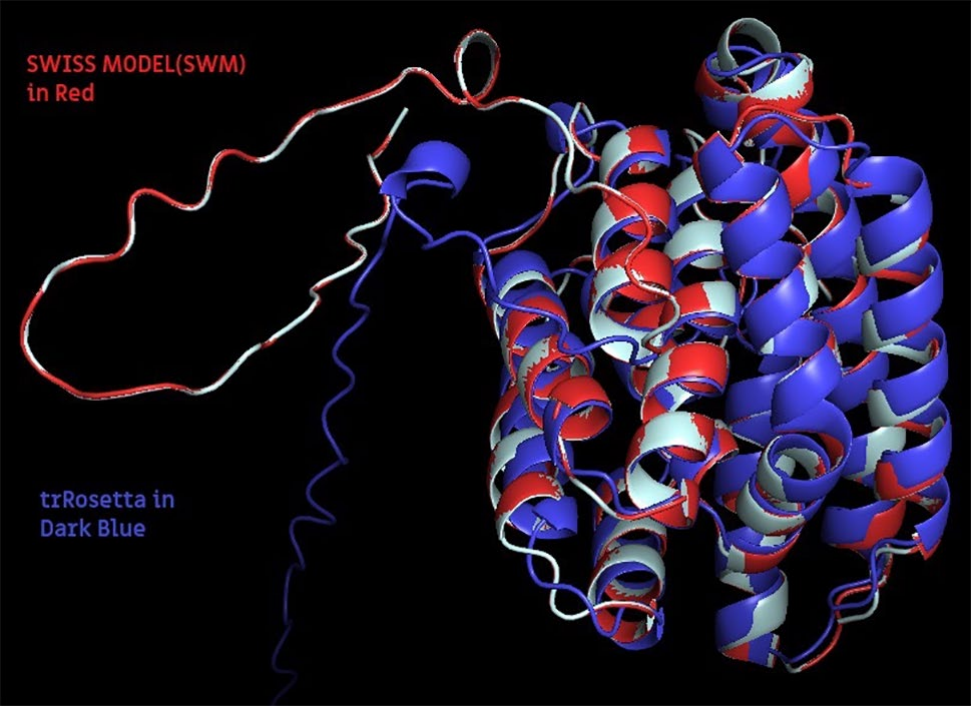
Modelling of *Bombyx mori* (XP_028029832) constructed through the utilization of two computational modeling servers: The Swiss Model Workspace Server and the trRosetta server. The protein native structure is presented in gray and the predicted model are shown in red and blue cartoons for Swiss Model and trRosetta, respectively.

To the best of our understanding, the three-dimensional configurations of proteins offer valuable insights into their molecular-level functionalities, thereby contributing to a diverse range of applications. An in-depth understanding of the operational mechanisms of protein complexes and networks requires a thorough depiction of their interactions and the overarching quaternary structure^27^. In instances where the three-dimensional structures of a protein and its binding partners are accessible or can be accurately modeled, the utilization of docking methods becomes feasible. These methods enable the generation of a three-dimensional model of the complex by leveraging the geometric and physicochemical complementarity of the interacting molecules. As noted in the preceding section, the FTIR and WAXD results demonstrate that sugar alcohols can alter the crystalline structure of silk fibroin films by promoting β-sheet formation. Consequently, it is conceivable that the random coil, alpha helix, and β-turn structures of silk fibroin may transition into β-sheet conformation. Specifically, the intra- and intermolecular interactions of silk fibroin could be influenced by sugar alcohols, facilitating the formation of stable β-sheet structures. In our previous investigation, ^1^ it was demonstrated that the incorporation of 30%wt glycerol led to a significant increase in β-sheet content. This implies that glycerol plasticizer supplementation enhances β-sheet formation in SF films while reducing the presence of random coil and helical structures in silk scaffolds. Therefore, it is hypothesized that the protein light chains, especially random coil and alpha-helix structures are primarily interacted with by sugar alcohols, prompting an exploration of the interaction between fibroin light chains and ligands that mimic these structures in silk scaffolds. In this present study, the three-dimensional structure of silk fibroin obtained from *Bombyx mori* was generated by employing two computational modeling servers: (1) The Swiss Model Workspace Server and (2) The trRosetta server, and the results are shown in Fig. 5.

The validation of the computational model, performed by evaluating each server algorithm with the AlphaFold accession number P21828 as a template, demonstrates a significant similarity in protein structure for both models. This is further confirmed by a high-quality protein–protein docking, as evidenced by a low root mean square deviation (RMSD), specifically 0.073 Å for Swiss Model and 1.036 Å for trRosetta. Therefore, the accurate prediction of protein structures was achievable through the utilization of the model algorithm, with particular emphasis on the effectiveness of the Swiss Model. The ascertained three-dimensional structure of silk fibroin is crucial for elucidating its plausible interactions with incorporated sugar alcohols. This structure will function as the designated protein framework to assess the binding interactions between the silk fibroin and sugar alcohols via computational docking.

**Figure 6:**
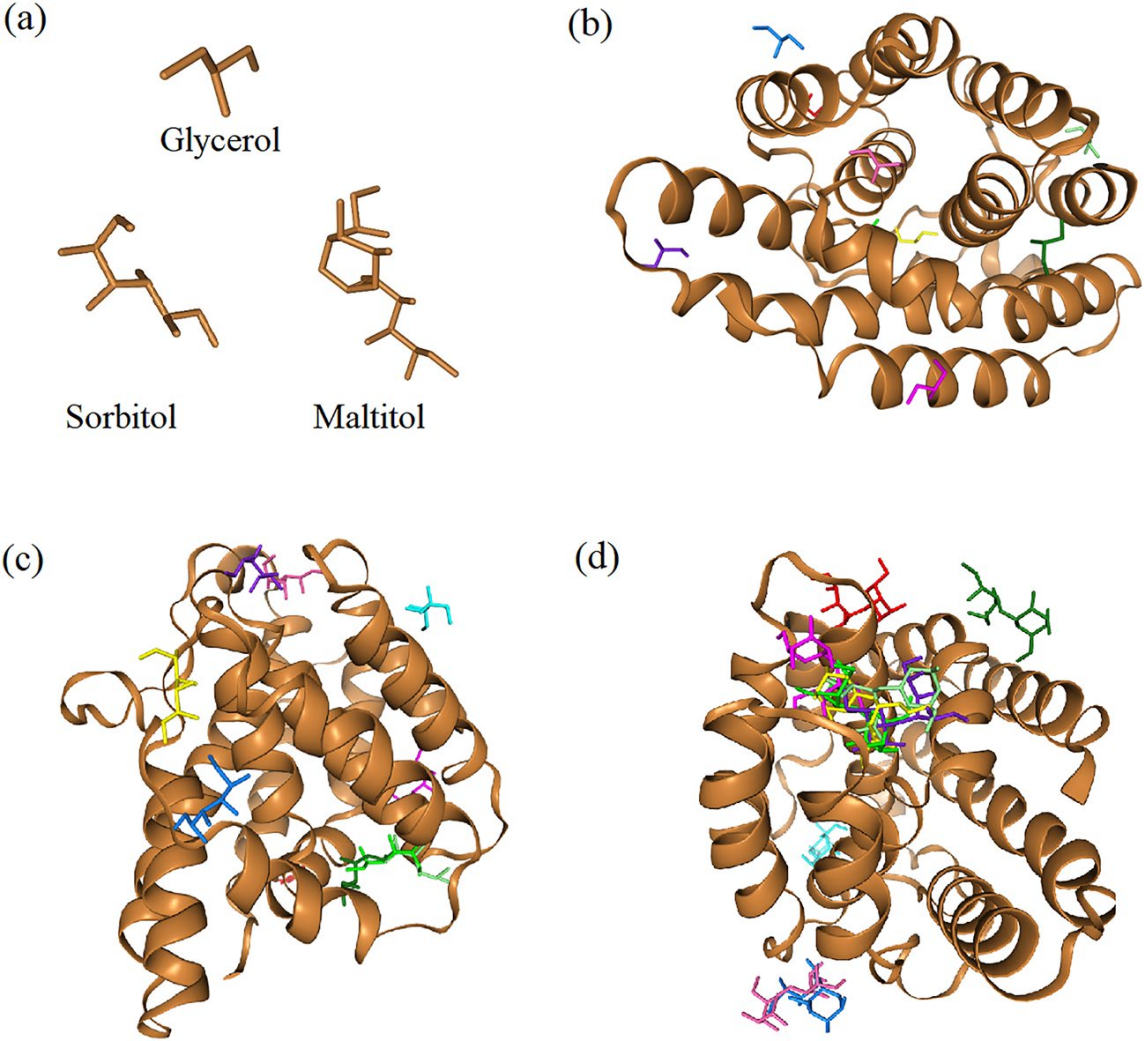
Structures of the incorporated sugar alcohols (**a**), and the molecular docking results of these sugar alcohols (ligands), presenting the superposition of ligands within the protein–ligand complex for glycerol (**b**), sorbitol (**c**) and maltitol (**d**).

The results obtained through computational docking analysis, conducted using the HDOCK server, evaluating the interaction between the fibroin light chain and ligands, as well as the self-interaction among ligands, are depicted in both Fig. 6 and Table 1. In the context of small size, glycerol molecules exhibit facile integration with polymer chains (Fig. 6b, Supplementary Fig. S3 online). On the contrary, molecules with larger sizes, denoted by higher molecular weights, demonstrate difficulty in merging with the host polymer at a molecular level. Notably, it was observed that sorbitol and maltitol, featuring larger molecules, did not achieve as effective fusion with silk fibroin chains as glycerol did, and their molecular aggregation was evident (Fig. 6c, d, Supplementary Figs. S4, S5 online) . Furthermore, this observation is substantiated by the docking scores, indicating that glycerol-glycerol exhibited the lowest docking score, followed by sorbitol-sorbitol and maltitol-maltitol, respectively (Table 1). This hierarchy suggests a potential for self-aggregation among sugar alcohols in the order of maltitol > sorbitol > glycerol. Similarly, the docking scores revealed that the binding between silk fibroin and maltitol exhibited a higher plausibility, followed by silk fibroin-sorbitol and silk fibroin-glycerol, respectively. Besides, the ligand rmds values suggest that the alignment of predicted ligand conformations with the expected or native configurations in the context of protein–protein interaction is acceptable^28^. Conclusively, the substantial size and intricate hydrogen bonding characteristics of sugar alcohols play a pivotal role in shaping their interactions within silk fibroin matrices. These interactions have discernible effects on phenomena such as diffusion and the overall structural integrity. In alignment with the findings outlined in the preceding section regarding WAXD results, the modeling outcomes confirm that the incorporation of larger sugar alcohol molecules with silk fibroin chains was comparatively less efficient than that of glycerol. Consequently, the transition from a random coil to α-helices, marking an unstable intermediate phase in the development of stable β-sheet structures, was observed infrequently. The results obtained also align well with experimental data from FTIR, WAXD and gel fraction analysis. Consequently, computational modeling proves to be a valuable tool for researchers, enabling them to predict the properties of silk fibroin materials by analyzing their conformations after interaction with additives such as alcohols, plasticizers, or nucleating agents, without the need for complex experiments.

**Table 1:** Results of modeling performance, illustrating the docking score, and ligand root mean square deviations (rmds) for both fibroin light chain-ligand and ligand-ligand computational docking.

Receptor-ligand	Docking score	Ligand rmds (Å)
Glycerol-glycerol	− 4.15	6.45
Sorbitol-sorbitol	− 8.19	7.30
Maltitol-maltitol	− 12.98	6.82
Silk fibroin-glycerol	− 72.0	10.62
Silk fibroin-sorbitol	− 95.8	17.89
Silk fibroin-maltitol	− 130.96	12.76

These results were obtained through the utilization of the HDOCK server.

### Ozone treatment of silicone

**Figure 7:**
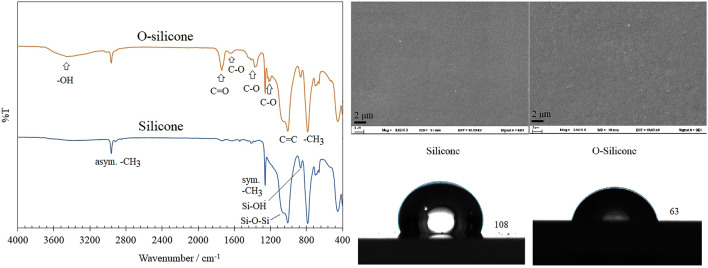
Fourier transform infrared (FTIR) spectra, scanning electron microscopy (SEM) micrographs depicting surface characteristics, and measurements of water contact angles for both untreated silicone and silicone subjected to UV/Ozone treatment (O-silicone).

One potential application of the silk fibroin films explored in this study involves their use as a hydrophilic coating for plastic medical devices, such as silicone urinary catheters. Poly(dimethylsiloxane) (PDMS) stands out as the predominantly employed silicone, characterized by pendant functionalities around the Si atom consisting of methyl groups. The incorporation of two stable methyl groups in each repeat unit affords PDMS high chemical resistance and a low surface energy. PDMS has been utilized extensively in the mentioned applications owing to its widespread availability, relatively economical nature, biocompatibility, and chemical inertness^29^. Numerous technological applications require PDMS surfaces that can be tailored to bind various chemical moieties. In the current investigation, the hydrophobicity of the silicone surface can be diminished through ozone treatment, commonly referred to as ozonation. The silicone surfaces are altered through a UV/ozone (UV/O) process in this approach, which is favored as it does not necessitate costly or intricate instrumentation or procedures. Moreover, the process can be conducted at room temperature^30,31^. Typically, within the UV/O process, ozone generated in situ reacts with the methyl-silicone (Si−CH_3_) groups on the silicone backbone, transforming them into (Si−O−) and consequently creating a hydrophilic (SiO_x_) layer on the silicone surface^32^. The alteration in the chemical structure of silicone following ozonation is evident through the FTIR spectra presented in Fig. 7.

The prominent alteration in the chemical structure of silicone upon exposure to UVO is manifested by the broadening and heightened peak intensity of R−OH (3130 − 3680 cm^−1^). While there are no apparent changes in the Si−OH signal at 865 cm^−1^ or the Si−O−Si signal at 1057 cm^−1^, an improvement in the hydrophilicity of silicone is anticipated. This expectation is supported by the contact angle values (Table 2), which clearly indicate an enhancement of the surface polarity^33^. This implies that the silicone surface undergoes efficient conversion to a material resembling silica, attributed to the removal of organic groups and the condensation of silanol groups with each other. Signals observed at 1740 cm^−1^ (C=O) and 1635 cm^−1^ (hydrogen-bonded carbonyl), as well as 1365 and 1215 cm^−1^ (C−O stretching), were attributed to the introduction of more hydrophilic moieties and the removal of carbon-containing groups from the surface of the sample. The production of these oxygen-containing species enhances the surface free energy and diminishes hydrophobicity^33^. Furthermore, owing to the remarkable stability of methyl groups on the silicone backbone, all signals associated with−CH_3_ (2962 cm^−1^, asymmetric−CH_3_ stretching; 1260 cm^−1^, symmetric−CH_3_ deformation; 1007 cm^−1^, C=C twist and=CH_2_ wagging; 784 cm^−1^, −CH_3_ rocking) display minimal changes with increasing UVO treatment. The obtained results align well with those documented in the existing literature^29,34^. SEM micrographs illustrating surface characteristics of silicone treated with UV/O (O-silicone) were additionally acquired to briefly examine the impact of UV/O treatment on surface morphology. The findings indicate that O-silicone displays surface profiles with increased roughness when compared to untreated silicone. This could be attributed to the plausible removal of organic contaminants through non-destructive etching during the UVO treatment process. Conclusively, UVO treatment emerges as a straightforward and efficacious approach for enhancing the hydrophilicity of silicone surfaces.

**Table 2:** Water contact angle (WCA) and cross-cut adhesion rating of bare silicone, silicone subjected to UV/Ozone treatment (O-silicone), and O-silicone coated with pure silk fibroin films (SF) and O-silicone coated with silk fibroin films integrating sugar alcohols, specifically glycerol (SFG), sorbitol (SFS), and maltitol (SFM).

Sample name	WCA /degree	Cross-cut adhesion rating
Silicone	108 ± 8	No data
O-Silicone	63 ± 2	No data
SF	44 ± 3	5B
SFG	26 ± 4	5B
SFS	42 ± 4	5B
SFM	38 ± 3	5B

### Physicochemical properties of silk firoin coatings

**Figure 8:**
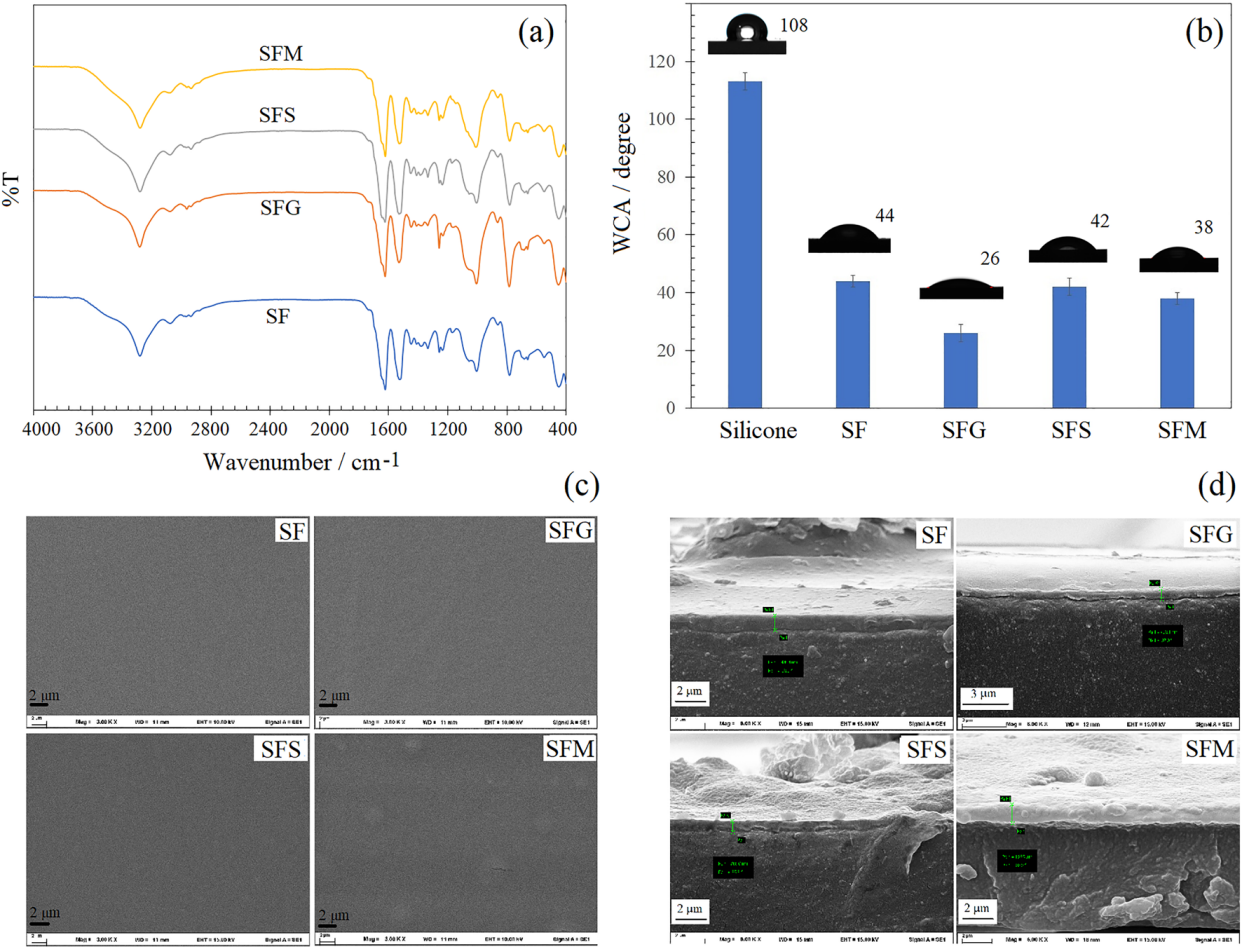
Fourier transform infrared (FTIR) spectra (**a**), water contact angle (**b**), surface (**c**) and cross-sectional (**b**) morphologies of ozonized silicone surfaces coated with silk fibroin films. The figure provides a thorough examination of silicone surfaces coated with pure silk fibroin films (SF) and with those integrating sugar alcohols, specifically glycerol (SFG), sorbitol (SFS), and maltitol (SFM).

To assess the efficacy of employing silk fibroin films as a hydrophilic coating for silicone surfaces, an in-depth examination of physicochemical properties, including chemical structure, surface and cross-sectional morphologies, water contact angle, and cross-cut adhesion of the coated silicone surface, was conducted. In Fig. 8a, the FTIR spectra of silicone surfaces coated with both pure silk fibroin films and those incorporating sugar alcohols reveal no discernible difference compared to silk fibroin films alone. This observation suggests that the process of coating silk fibroin films onto the silicone surface was accomplished successfully, without inducing any alterations in the chemical structure of the silk fibroin films. We conducted an evaluation of the surface wettability of pristine silicone, comparing it to UVO-treated silicone coated with pure silk fibroin films and silk fibroin films incorporating sugar alcohols. This assessment was carried out through static contact angle measurements. In Fig. 8b and Table 2, UVO-treated silicone, when coated with SF, SFG, SFS, and SFM, demonstrates considerable reductions in contact angle, amounting to 59.3%, 75.9%, 61.1%, and 64.8%, respectively, as compared to bare silicone. The data analysis indicates that SFG has the most notable impact on reducing the contact angle, attributed to the heightened hydrophilicity induced by the presence of glycerol molecules. As previously discussed, glycerol, characterized by a compact three-carbon structure and three well-coordinated hydroxyl groups, facilitates improved diffusion between protein chains. On the contrary, sorbitol, with its larger six-carbon structure and hydroxyl groups arranged in diverse directions, poses an obstacle to effective diffusion. This characteristic contributes to the diminished permeability of sorbitol within the silk protein structure. Furthermore, sorbitol molecules have the capacity to engage in hydrogen bonding interactions among themselves, giving rise to an aggregated structure. These interactions restrict the interaction with water molecules. Hence, the water contact angle (WCA) of SFS (42 ± 4°) was greater than that of SFG (26 ± 4°). Maltitol, with its twelve-carbon structure, exhibits a significant abundance of equatorial−OH groups in comparison to sorbitol. This is attributed to a higher density of hydroxyl groups available for intermolecular hydrogen bonding between maltitol and water molecules, distinguishing it from sorbitol. Consequently, WCA of SFM (38 ± 3°) surpassed that of SFS (42 ± 4°).

SEM images depicting UVO-treated silicone coated with SF, SFG, and SFS reveal a uniform surface morphology (Fig. 8c). It is apparent that the coating process does not exhibit substantial alterations in the physical characteristics of the surface morphology or induce any discernible degradation in silk fibroin films. The coating thickness was evaluated using cross-sectional SEM images (Fig. 8d). All coatings exhibited strong adhesion, forming a thin and dense layer on the surface of ozonized silicone. The measured thicknesses were 1023 ± 53 nm for SF, 726 ± 26 nm for SFG, 802 ± 52 nm for SFS, and 839 ± 67 nm for SFM. Despite the presence of small amount of air bubbles on the surface of UVO-treated silicone coated with SFM, the cross-cut adhesion rating for all samples consistently registers at class 5B. Silk fibroin film surface adhesion was assessed on a silicone substrate using the cross-cut adhesion technique. The adhesion characteristics of the prepared films are presented in Table 2. The cross-cut adhesion outcomes were evaluated using a scale ranging from 0 to 5, where a rating of 5 signifies outstanding adhesion, and 0 indicates complete delamination^35^. No square specimen was observed to detach due to the adhesive tape for any of the formulated silk fibroin films. This observation suggests that the films demonstrated exceptionally strong adhesion to silicone. The robust adhesion observed between the silk fibroin film and the ozonized silicone surface is attributed to the establishment of hydrogen bonding interactions. These interactions primarily involve the hydrophilic groups, such as OH groups present on the ozonized silicone surface, and NH_2_ groups in silk fibroin films, along with OH groups in sugar alcohols. In conclusion, the silk fibroin films suggested in this study have the potential to serve as coatings for ozonized silicone surfaces and are deemed suitable for such applications.

## Conclusions

This investigation unveiled the potential for modifying the structure of silk fibroin films through the utilization of sugar alcohols possessing diverse chemical structures, carbon atoms, and hydroxyl groups. The extraction of silk fibroin was achieved through a conventional degumming procedure, and the production of well-defined silk fibroin films was accomplished via solution casting. The structure of derived silk fibroin underwent characterization through FTIR and X-ray. The conformational structural content of silk fibroin films was examined through curve-fitting of the Amide I region using a Python library.The accurate prediction of protein structures was achievable through the utilization of the model algorithm, with particular emphasis on the effectiveness of the Swiss Model. Furthermore, a case study investigating the application of silk fibroin films as a surface coating for silicone was conducted.

The findings substantiate that sugar alcohols featuring diverse chemical structures can indeed modify the film characteristics. While all the films exhibited the notable transparency inherent to silk fibroin films and their ability to absorb UV light, the UV-absorption properties of silk fibroin films were accentuated with the incorporation of glycerol. FTIR analysis affirmed that, concerning their effectiveness as sugar alcohol plasticizers in enhancing the β-sheet structure, the hierarchy is as follows: glycerol > sorbitol > maltitol. These results align consistently with those obtained from WAXD. The outcomes highlight distinctive characteristics related to varied and manageable transitions in silk structure, and straightforward fabrication (achieved through a one-step film casting process without additional treatments). These attributes enhance the film's utility in coating applications. Furthermore, the computer simulation validated the interaction patterns between sugar alcohol molecules and silk protein chains, as well as the intermolecular interactions among sugar alcohol molecules. This observation is contingent upon the size and quantity of hydroxyl groups present in sugar alcohols. Conclusively, a larger sugar alcohol demonstrates a more negative docking score, indicative of a heightened potential for binding between silk protein chains. This extends to interactions among the sugar alcohol molecules themselves. Therefore, the size and hydrogen bonding characteristics inherent in sugar alcohols play a crucial role in influencing their interactions within silk fibroin matrices. Convincingly, computational modeling emerges as an invaluable tool for researchers. It enables the prediction of silk fibroin material properties by analyzing their conformations following interactions with additives, thereby avoiding the necessity for complex experimental procedures.

The adhesion of all silk fibroin coatings onto ozonized silicone surfaces was excellent, given that ozonation enhances the hydrophilicity of silicone surfaces. The cross-cut adhesion rating consistently achieves class 5B for all samples. Furthermore, the coating process does not introduce significant changes to the physical characteristics of surface morphology nor does it induce noticeable degradation in silk fibroin films. UVO-treated silicone, when coated with SF, SFG, SFS, and SFM, exhibits considerable reductions in contact angle ranging from 59.3% to 75.9% compared to untreated silicone. This ensure that the modified silk fibroin films are suitable for application as hydrophilic coatings for medical devices specifically crafted from silicone materials.

### Supplementary Information

Supplementary Information.

## Data Availability

The authors declare that the data supporting the findings of this study are available within the paper and its Supplementary Information files. Should any raw data files be needed in another format they are available from the corresponding author upon reasonable request.
