# Supplementary material for: Tailoring silk fibroin hydrophilicity and physicochemical properties using sugar alcohols for medical device coatings
Source: Sci Rep. 2024 Jun 14;14:13781. doi: 10.1038/s41598-024-64450-5 (PMC11178791; doi:10.1038/s41598-024-64450-5)
Supplement: Supplementary file 1 — Supplementary Information. [file 41598_2024_64450_MOESM1_ESM.docx]

**Supplementary Information**

**Tailoring Silk Fibroin Hydrophilicity and Physicochemical Properties Using Sugar Alcohols for Medical Device Coatings**

Supranee Kaewpirom^a,*^, Sarayoot Piboonnithikasem^b^ Pongsathorn Sroisroemsap^a^, Sittichai Uttoom^a^, Siridech Boonsang^c^

^a^Department of Chemistry, Faculty of Science, Burapha University, Chonburi 20131,

Thailand

^b^Department of Medical Science, National Institute of Health, Ministry of Public Health, Nonthaburi 11000, Thailand.

^c^Department of Electrical Engineering, Faculty of Engineering, King Mongkut's Institute

of Technology Ladkrabang, Bangkok 10520, Thailand.

E-mail: kaewpiro@go.buu.ac.th; Fax: +66 38 393 494; Tel: +66 38 103 066

**Table S1** Chemical structure, numbers of C atom, and numbers of OH group present in the sugar alcohols incorporated into silk fibroin films.

| Sugar alcohol | Chemical structure | numbers of C atom | number of OH groups | Name of silk fibroin films containing sugar alcohols |
| --- | --- | --- | --- | --- |
| Glycerol  (92.09 g/mol)  Solubility in water: miscible | 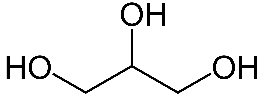 | 3 | 3 | SFG |
| Sorbitol  (182.17 g/mol)  Solubility in water: 2,350 g/L | 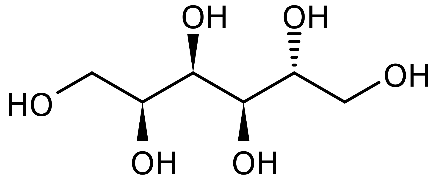 | 6 | 6 | SFS |
| Maltitol  (344.31 g/mol)  Solubility in water: 1,750 g/L | 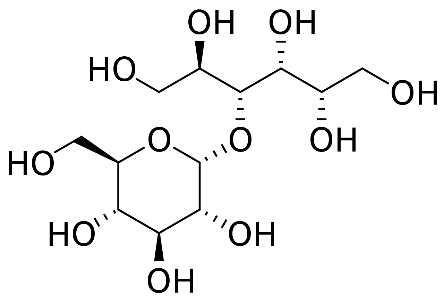 | 12 | 9 | SFM |

**Figure S1** Ultraviolet-visible spectroscopy (UV/VIS) spectra of silk fibroin films (a,b), indicating significant transparency in the visible spectrum and distinct UV absorption characteristics.

**Figure S2** Relative β-sheet content of silk fibroin films without alcohol treatment (SF), and treated with glycerol (SFG), sorbitol (SFS), and maltitol (SFM).


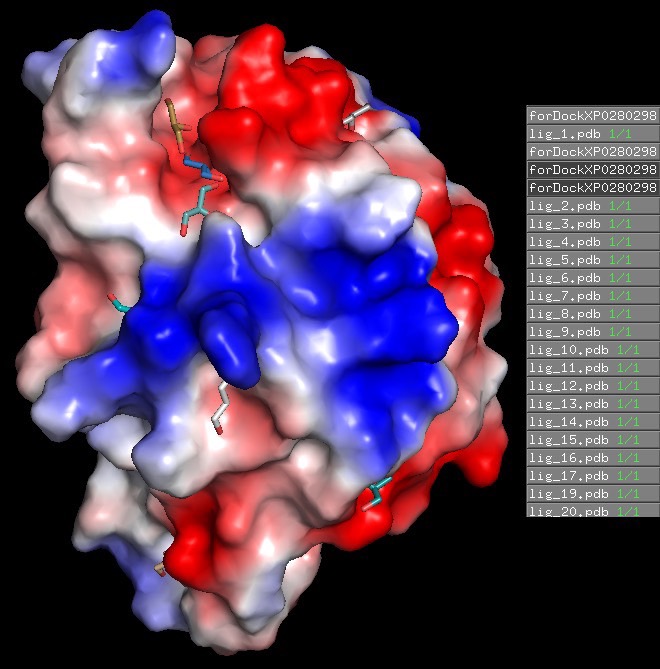


**Figure S3** The results obtained through computational docking analysis, conducted using the HDOCK server, presenting the superposition of ligands within the protein-ligand complex for glycerol.


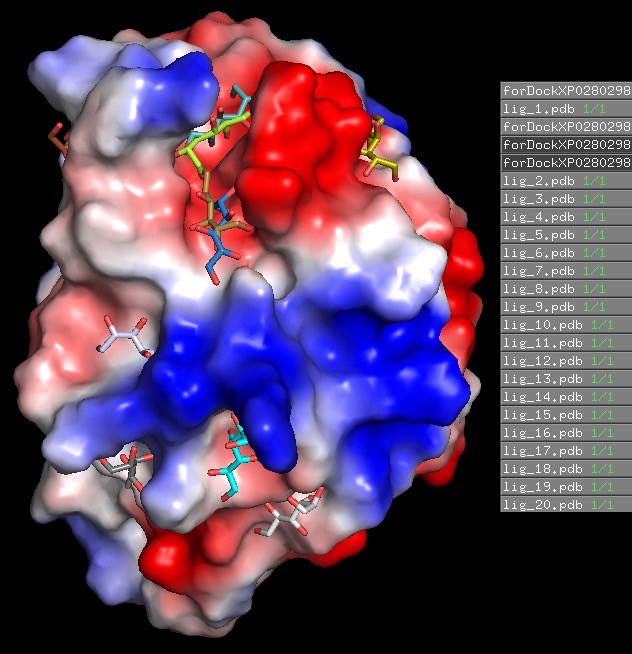


**Figure S4** The results obtained through computational docking analysis, conducted using the HDOCK server, presenting the superposition of ligands within the protein-ligand complex for sorbitol.


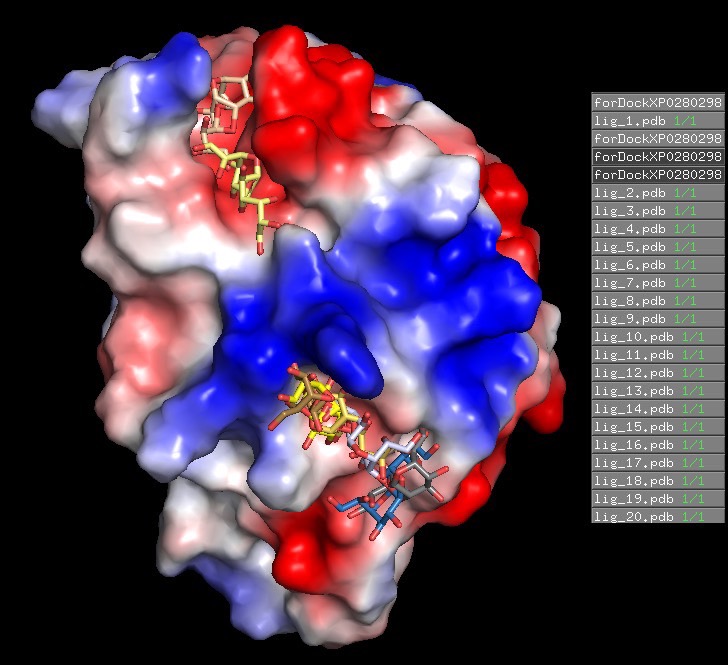


**Figure S5** The results obtained through computational docking analysis, conducted using the HDOCK server, presenting the superposition of ligands within the protein-ligand complex for maltitol.
